# Topical treatment of oral chronic graft-versus-host- disease in hematopoietic stem cell transplant recipients: A systematic review

**DOI:** 10.4317/jced.60138

**Published:** 2023-05-01

**Authors:** Livia Haas, Marta Cruz-Pamplona

**Affiliations:** 1 Degree in Dentistry. Faculty of Health Sciences. Universidad Europea de Valencia. Spain; 2 Degree in Dentistry. Master in Oral Medicine and Surgery. Faculty of Medicine and Dentistry. University of Valencia. Spain

## Abstract

**Background:**

Oral graft-versus-host disease (GVHD) is a common complication of hematopoietic stem cell transplantation. This study systematically reviewed Randomized Controlled Trials (RCTs) with the objective to investigate the effectiveness and side effects of topical agents used for the treatment of oral GVHD.

**Material and Methods:**

The Preferred Reporting Items for Systematic Review and Meta-Analysis (PRISMA) guidelines were followed to perform this study. An electronic search of four databases was conducted. RCTs published between January 2011 and March 2022 were included that were carried out on hematopoietic stem cell transplant recipients receiving topical treatment for oral GVHD. The Critical Appraisal Skills Program (CASP) standard checklist for RCTs was used for the bias risk evaluation.

**Results:**

Five RCTs were included for the qualitative synthesis of results. Two RCTs were linked to a certain risk of bias. Budesonide caused the highest overall treatment response. Malic acid, clobetasol, and dexamethasone increased resting salivary flow rates. Curcumin in orabase shows similar results to corticosteroid treatment. Adverse effects were observed in populations receiving budesonide, dexamethasone, clobetasol, and tacrolimus. Most frequent adverse effects were burning sensations, fungal infections, and gastrointestinal disorders, but none of them were severe.

**Conclusions:**

Given the small number of RCTs performed and the heterogeneity of the different study designs, it is difficult to draw direct comparisons. Malic acid appears to be effective for the treatment of graft-versus-host disease-induced xerostomia. Budesonide had the highest overall response rates but was also associated with the highest number of adverse effects. Further research is needed to manifest those findings.

** Key words:**Hematopoietic stem cell transplant, oral graft-versus-host disease, topical treatment.

## Introduction

Hematopoietic stem cells are multipotent cells and are responsible for the generation of all functional hematopoietic lineages (1). Hematopoietic stem cell transplants (HSCTs) aim to counteract problems related to the inappropriate functioning of the hematopoietic system, like hematologic malignancies, select solid tumors, nonmalignant conditions, and severe immunologic deficiencies (1-4). The rationale of HSCTs is to achieve a broad lymphoablation that allows an initial breakdown of the immunological memory repertoire. As a result, the hematopoietic and thus the immune system is regenerated, which enables an immunological renewal (3). Due to the severe immunosuppression, as well as the rigorous conditioning regimen applied in oncologic patients, the patients might suffer severe complications (5). The major lethal complication of HSCT is graft-versus-host disease (GVHD), an immunological disorder in which the donor’s lymphocytes attack the healthy recipient’s tissues (5-7). GVHD is a multisystemic disorder affecting several organs. The skin is the main manifestations site, however many manifestations present itself in the oral cavity (5,7-9). Common oral manifestations include erythema, erosions, ulcers, lichenoid lesions, xerostomia, and pain (8,10). In some cases, mucoceles and mucosal atrophy have also been observed (7). The first-line therapy for GVHD are systemic corticosteroids. However, due to their associated secondary effects like osteoporosis, and avascular necrosis, topical alternatives are under current investigation (10,11). For oral GVHD, common corticosteroid solutions or gels are dexamethasone, budesonide, clobetasol, prednisolone, and triamcinolone (10,12,13). Another alternative under current investigation is gel rich in platelets (14,15). A non-steroidal option includes tacrolimus (12,16). Non-pharmacological options comprise different types of phototherapies, like psoralen ultraviolet-A, UV-B therapy, photobiomodulation therapy, and carbon dioxide laser therapy (12,17,18). Even though there are many options available, there is still no consensus on the most effective option, nor standardized guidelines. This review aims to provide a systematic approach of literature including RCTs investigating the efficacy and possible side effects of topical agents used for the treatment of oral GVHD.

## Material and Methods

- Protocol and focused question:

This review has been registered at Prospero (CRD42022315537). A systematic review was conducted including RCTs to compare different topical treatment agents. The PRISMA (Preferred Reporting Items for Systematic Reviews and Meta-Analyses) guidelines were followed (19). The following question was developed according to the population, intervention, comparison, and outcome study design. What is the most effective topical treatment for oral graft-versus-host disease in hematopoietic stem cell transplant recipients? 

- Selection criteria:

Only RCTs published between January 2011 and March 2022 in languages English, German, or Spanish were included. The studies had to be performed *in vivo* on humans with clear description of the topical therapy used and its method of application. Studies comprised of less than 10 participants, studies focusing on prophylactic measures or systemic treatment of oral GVHD, and studies including the treatment of extra-oral manifestations were excluded.

- Search strategy:

An electronic search was conducted via MEDLINE Complete, PubMed, Scopus, and Cochrane central Register of Controlled Trials. The following Medical Subject Heading (MeSH) and text words were used: (“Graft vs Host Reaction” OR “Graft vs Host Disease” OR “GVHD” OR “Graft-versus-host disease” OR “Graft versus host disease” OR “Graft-versus-host reaction” OR “Chronic oral graft versus host disease”) and combined with the Boolean term “AND” (“Topical” OR “Local treatment” OR “Oral” OR “Mouthwash” OR “Buccal” OR “Topical treatment” OR “Topical corticosteroids” OR “Topical administration” OR “Spray” OR “Gel” OR “Topical therapy”). Manual search of reference list was performed to identify additional articles.

- Screening methods and data abstraction:

Two independent reviewers (LH and MCP) performed the search. After removal of duplicates, the titles and abstracts were scanned for eligibility. Full text analysis was performed of the articles considered eligible and inclusion and exclusion criteria were applied. In case of disagreement regarding inclusion, discrepancy was resolved by mutual consensus of all reviewers. The following data was extracted: author, year of publication, country, study type, sample size, population gender and age, manifestations at baseline, treatment design, length of study, treatment response, and side effects.

- Risk of bias:

The Critical Appraisal Skills Program (CASP) standard checklist for RCTs was used to evaluate the potential risk of bias (20). The checklist is comprised of four segments referring to basic study design, methodology, results, and discussion. These segments were evaluated by two reviewers (LH and MCP) and an overall assessment of risk of bias was performed ranging from low, high, or certain risk of bias.

## Results

**Figure 1:**
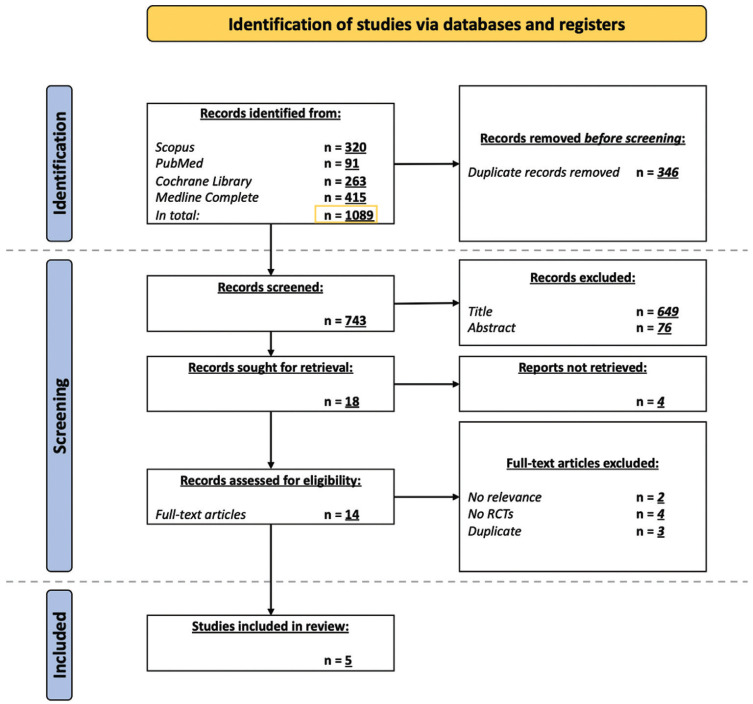
Flow-chart of the search carried out in the four databases.

- Study selection:

From the 1089 studies retrieved during the search, 14 were considered eligible and according to the inclusion and exclusion criteria, a total of 5 studies were included: two Randomized Double-Blind Clinical Trial (21,22), an Open, Randomized, Multicenter Trial (23), an Open-Label Phase II Randomized Trial (24), and a Randomized Clinical Trial (25). Figure 1 shows the identification, screening, and inclusion of the studies included in this systematic review.

- Characteristics of included studies:

The studies were published between 2012 and 2019, involving a total of 157 patients of which 140 were evaluated at baseline and at the end of the study. The studies were carried out in Germany/ Israel, Brazil, United States of America, Iran, and Italy. The mean age of the participants varied from 35.8 to 62.5 and the sex ratio was male dominant. Further characteristics are shown in Table 1. Oral manifestations involved in cGVHD were erythema, atrophy, ulcer, lichen, hyperkeratosis, pseudomembrane, edema and mucocele, appearing as a mucus cyst on the soft palate, on the labial and buccal mucosa, and xerostomia. Oral manifestations linked to GVHD diagnosis was done on different parameters across the included studies: World Health Organization (WHO) toxicity oral/ gastrointestinal, modified Oral Mucosal Rating Scale (mOMRS), Oral Mucositis Assessment Scale (OMAS), NIH oral cavity severity score, mucosal score, and oral symptoms score, Dry Mouth Questionnaire (DMQ), sialometry, various Visual Analogue Scales (VAS), and biopsies (21-25).

**Table 1:**
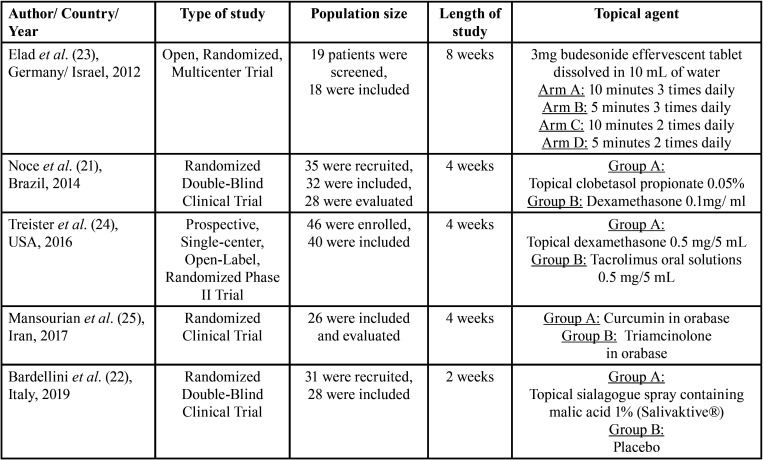
Characteristics of the included studies.

**Figure 2:**
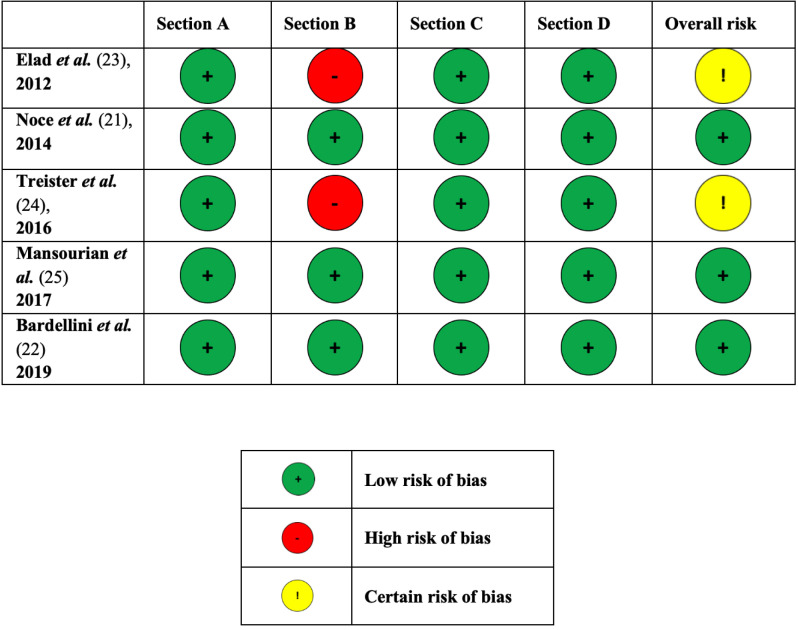
Estimated risk of bias of the included randomized controlled trials.

- Risk of bias:

Figure 2 shows the estimated risk of bias. Two studies were considered at certain risk of bias due to the missing of blinding of patients and interventionists (23,24).

- Synthesis of the results:

The different topical therapeutics used in the studies included were, topical dexamethasone, topical budesonide, malic acid, topical clobetasol, topical tacrolimus, triamcinolone in orabase, and curcumin in orabase. Elad *et al*. (23) observed median relative reduction in the mOMRS of 70%, of 69% in the OMAS, and of 61% in the WHO toxicity scale gastrointestinal/ oral. The rate of objective response which was defined as more than 50% compared to baseline using the mOMRS was not significantly different among the 4 study arms. In the study of Noce *et al*. (21) there was a reduction of 3.0 (53.9% of cases) in the mOMRS, reduction of 2.1cm in the VAS symptomatic response, reduction of 0.10cm in the VAS xerostomia score, and n increase of 0.11mL/min of resting salivary flow rate (SFR) in the clobetasol group. For the dexamethasone group, reduction of 1.0 (26.7% of cases) in the mOMRS, reduction of 1.4cm in the VAS symptomatic response, and a reduction of 1.75cm in the VAS xerostomia could be observed. The median reduction in mOMRS total score was significantly higher in the clobetasol group than the reduction observed in the dexamethasone group (*p*=0.03). Also, the median reduction in the symptomatic response (VAS) was significantly better for the clobetasol group than for the dexamethasone group (*p*=0.02). The median VAS xerostomia scores were significantly improved in patients in the dexamethasone group (*p*=0.04) but not in the clobetasol group (*p*=0.06). A significant increase in the median SFR in the clobetasol group was noted (*p*=0.01) but no significant differences in SFR were observed in the dexamethasone group (*p*=1.00). In the study conducted by Treister *et al*. (24), for the dexamethasone group a sensitivity response in 58% was observed. 69% achieved an overall response. The OMS response was 8%, and the NIH Oral Cavity Severity Score response 50%. The tacrolimus arm was closed early due to a lack of activity in the sensitivity response with 21% only. Overall response was observed in 50%, 36% in the OMS response, and 14% responded to the NIH Oral Cavity Severity Score response. Mansourian *et al*. (25) observed a mean severity reduction of 4.11±1.04mm2 in the curcumin group and a reduction of 1.93±0.37 in the VAS pain severity at day 14, and 3.77±0.66 at day 28. In the triamcinolone control group, mean severity reduction was 4.23±1.49mm2. Reduction of 4.46±0.37 according to the VAS pain severity was stated at day 28. There was no significant difference of the alleviated severity between the two groups (*p*=0.052). Also, the severity of the pain at the baseline (*p*=0.287) and day 28 (*p*=0.687) was not significantly different between the two groups. In the study conducted by Bardellini *et al*. (22) the DMQ scores increased by 2.2 points. The unstimulated SFR increased by 0.09±0.02mL/min. A significant increase of *p*<0.05 was observed in the DMQ scores, as well as in the SFR with *p*<0.05. Table 2 shows the different unstimulated salivary flow rates pre- and post-intervention.

**Table 2:**
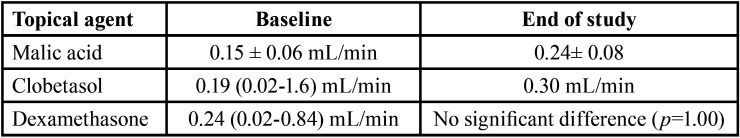
Unstimulated salivary flow rate in comparison.

**Figure 3:**
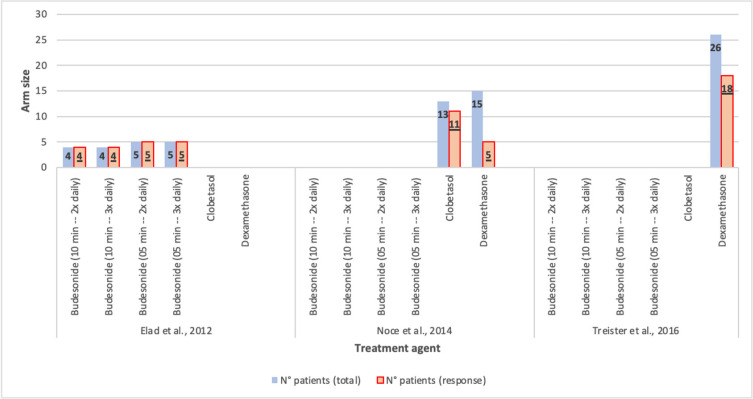
Comparison of overall response between patients receiving budesonide, clobetasol, and dexamethason.

Figure 3 shows the overall response of patients receiving budesonide, clobetasol, and dexamethasone. For the study carried out by Elad *et al*. (23), the improvement of 50% of the mOMRS was defined as objective response, for Noce *et al*. (21) the symptomatic response was taken into consideration, and for Treister *et al*. (24) the overall response described by the authors was used.

Reported adverse effects were gastrointestinal disorders, such as cheilitis and esophagitis, fungal infections like candidiasis, and nervous system disorder like taste alterations, burning sensations and oral cavity pain (21-25). Additional data is described in Table 3. Most adverse effect could be seen in the treatment with budesonide, where 44.4% of the patients referred to side effects (23). 7.14% of the patients treated with clobetasol and 7.14% of the patients treated with tacrolimus solution developed adverse effects (21,24). Only 4.55% of the patients treated with topical dexamethasone reported side effects (21).

**Table 3:**
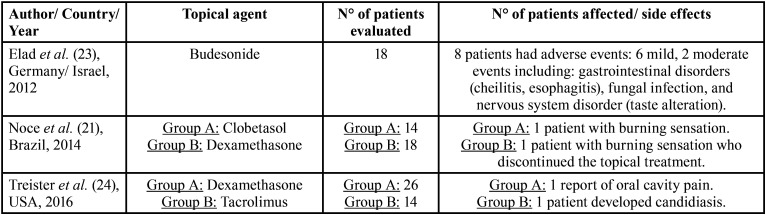
List of reported adverse effects.

## Discussion

There are only a few systematic reviews assessing the different topical agents for the treatment of oral GVHD. Albuquerque *et al*. (26) conducted a systematic review published in 2016 analyzing seven studies on the management of oral GVHD. They emphasized the need of high quality RCTs investigating the efficacy of treatment of oral GVHD to establish clinical guidelines. Elsaadany *et al*. (27) included six clinical trials focusing on the topical treatment with corticosteroids. According to their results, clobetasol, followed by budesonide showed promising clinical efficacy but due to the lack of RCTs, judging the efficacy and safety of the topical agents was a major limitation according to the authors. Sava *et al*. (28) carried out a systematic review on the topical treatment of oral manifestations of GVHD, focusing on topical corticosteroids only due to the lack of RCTs carried out on alternative agents.

- Most effective topical treatment:

In this systematic review, 5 RCTs were included, most of which had a small population size. It was evident, that there is a great degree of heterogeneity within the studies. Regarding the treatment of GVHD-induced xerostomia, Bardellini *et al*. (22) stated a significant increase in the DMQ score and unstimulated salivary flow rate. Malic acid shows some advantages over other acids tested in the past. Citric acid has been previously studied as sialagogue, however, due to its demineralizing effects on human dentin and subsequently increased risk of caries, its use has been repudiated (29). Most of the products containing high doses of acidic components are mainly associated with chewable consumption, which prolongs the contact of the product with the tooth surface and thus enhances the erosive action. Malic acid’s mechanism of action is linked to the dissociation of H+ in malic acid in water, hydronium ions formation and subsequent stimulation of salivary secretion aiding the dilution of acids in the oral cavity (30). Furthermore, the product tested by Bardellini *et al*. (22) contains xylitol, which counteracts the erosive action and the cariogenic potential. According to Noce *et al*. (21), the increase of resting SFR when applying clobetasol was rather unexpected. When comparing the overall response, the authors stated clobetasol was also more effective than dexamethasone which had a low response rate (21). In previous studies, when comparing the two agents in the treatment of oral lichen planus, topical clobetasol has proven to be more effective (31,32). In a study conducted by Wolff *et al*. (33) dexamethasone had a high response of 68.75% when used as topical treatment of oral GVHD. However, their participants received the topical agent up until 9 months. When comparing the response rates on the mOMRS, budesonide caused the highest response regardless of the different arms, followed by clobetasol, and dexamethasone. This finding was also confirmed by Sava *et al*. (28). The tacrolimus arm of Treister *et al*. (24) was closed early due to lack of activity in the sensitivity response. Effectiveness of topical tacrolimus has been assessed in several case reports and series, however, there is no larger sample size RCTs. In a study conducted by Mawardi *et al*. (16), a synergistic effect when combined with topical steroids was observed, however, it was not proven to be effective when administered on its own. Mansourian *et al*. (25) stated that for curcumin and triamcinolone in orabase, severity of oral involvement, as well as pain severity improved with no significant difference between both groups. Curcumin has long been used in traditional medicine for wound healing and pain relief. Recent studies have shown that it decreases the levels of TNF-α, IL-1ß and IFN-γ cytokines and thus exerts anti-inflammatory and antioxidative effects (34). Furthermore, it has been related to antibacterial, antifungal, antiviral, and disinfecting properties, which might be beneficial in the treatment of oral lesions in GVHD (35). However, further research is needed to manifest that thesis.

- Side effects:

Regarding the side effects, budesonide appears to cause the most adverse effects (23). None of them were considered severe. It also must be taken into consideration, that the side effects reported, such as affectation of the GI tract, fungal infections, etc., are also common characteristics of complications of GVHD (5,36). Bardellini *et al*. (22) did not give any information on possible side effects leading to the assumption, that no unpleasant events were reported, however, a long-term follow-up study would be of interest, investigating the effect of malic acid on the dental enamel, as it was linked to erosive capacity in previous studies.

Limitations of this review include the use of four databases only during the search. Added to that, only articles published in English, German, and Spanish language were reviewed, leading to a possible exclusion of other relevant data. The low number of RCTs included in this review represents a major limitation when it comes to drawing conclusion on the efficacy of topical treatment on oral involvement of GVHD. Furthermore, an objective outcome was defined by the authors to compare the studies’ overall response. This bears risk of misinterpretation of the studies’ results, as well as bias. In the future, more RCTs should be carried out with a larger number of participants and over a longer period.

Various parameters should be considered, such as administered systemic treatment and conditioning regimen, and underlying diseases. Also, similar study designs would allow a better comparison between the studies, taking into consideration the time of intervention, as well as the assessment tools used for diagnosing and final evaluation. Furthermore, there is a need for standardization of systemic treatment regimen for HSCT-recipients, diagnostic methods, assessment tools for GVHD, and first-line topical therapy for the treatment of oral GVHD.

## Conclusions

To conclude budesonide showed the highest overall response, as well as the most adverse effects independently from the different administration protocols. Malic acid seems effective for the treatment of GVHD-induced xerostomia. More research is needed to manifest those findings.
